# A New Dyspnea Evaluation System Focusing on Patients’ Perceptions of Dyspnea and Their Living Disabilities: The Linkage between COPD and Frailty

**DOI:** 10.3390/jcm9113580

**Published:** 2020-11-06

**Authors:** Keiji Oishi, Kazuto Matsunaga, Misa Harada, Junki Suizu, Keita Murakawa, Ayumi Chikumoto, Yuichi Ohteru, Kazuki Matsuda, Sho Uehara, Kazuki Hamada, Shuichiro Ohata, Yoriyuki Murata, Yoshikazu Yamaji, Maki Asami-Noyama, Nobutaka Edakuni, Tomoyuki Kakugawa, Tsunahiko Hirano

**Affiliations:** 1 Department of Medicine and Clinical Science, Graduate School of Medicine, Yamaguchi University, Ube 755-8505, Japan; ymurata-ygc@umin.ac.jp; 2 Department of Respiratory Medicine and Infectious Disease, Graduate School of Medicine, Yamaguchi University, Ube 755-8505, Japan; kazmatsu@yamaguchi-u.ac.jp (K.M.); hara-da@yamaguchi-u.ac.jp (M.H.); relativity.theory135@gmail.com (J.S.); murakawakeita124@gmail.com (K.M.); chiku05@yamaguchi-u.ac.jp (A.C.); yohteru@yamaguchi-u.ac.jp (Y.O.); k0m1a2t8s1u1d2a1@gmail.com (K.M.); n010eb.mie@gmail.com (S.U.); khamada@yamaguchi-u.ac.jp (K.H.); j015ebponyou@gmail.com (S.O.); yyamaji@yamaguchi-u.ac.jp (Y.Y.); noyamama@yamaguchi-u.ac.jp (M.A.-N.); edakuni@yamaguchi-u.ac.jp (N.E.); tsuna@yamaguchi-u.ac.jp (T.H.); 3 Department of Pulmonology and Gerontology Graduate School of Medicine, Yamaguchi University, Ube 755-8505, Japan; tomoyukikakugawa@gmail.com

**Keywords:** dyspnea, PROMs, activity-limit, self-limit, frailty

## Abstract

Background: As much as there are unmet needs for brief frailty assessment in patients with chronic obstructive pulmonary disease (COPD), the lack of a simplified and comprehensive dyspnea evaluation system that focuses on the patients’ perceptions of dyspnea and their COPD living disabilities remains a major challenge. We developed patient-reported outcome measures for dyspnea-related behavior and activity limitation (PROMs-D), which consisted of the Activity-limit Dyspnea Scale (ADS) and Self-Limit Dyspnea Scale (SDS), while investigated the usefulness of PROMs-D in identifying frailty. Methods: We administered PROMs-D and frailty status evaluations in 128 outpatients. Results: We classified 30 (23.4%), 50 (39.0%), and 48 (37.5%) patients as robust, prefrail, and frail, respectively. There was a positive correlation between SDS and ADS (*ρ* = 0.67, *p* < 0.001), and both ADS and SDS had high accuracies for detecting frailty (AUC, 0.82 and 0.78, respectively). Moreover, a PROMs-D score that consisted of the sum of ADS and SDS was more effective in stratifying frailty (cutoff value, 2; AUC, 0.85; sensitivity, 60%; specificity, 95%). Conclusions: PROMs-D could be used as the first step for frailty screening in patients with COPD, and we propose the importance of capturing the troublesome nature of living behaviors due to dyspnea in daily clinical practice.

## 1. Introduction

Chronic progressive pulmonary diseases, including chronic obstructive pulmonary disease (COPD) and interstitial lung diseases (ILDs), should be managed according to symptom phases and activity limitations in individual patients [1,2,3,4]. Many previous studies have demonstrated that it is critical to capture the effects of dyspnea on living activities and when daily life becomes more restrictive [5,6,7,8]. Furthermore, living disabilities due to dyspnea could guide the introduction of pharmacological and non-pharmacological approaches, and severe daily life restrictions may provide an important starting phase for consultation on advanced care planning with patients and their families [9,10,11].

The nature of dyspnea is reliant on patients’ self-reported symptoms. However, many COPD patients self-limit their living behaviors to avoid experiencing dyspnea, and therefore, underreport the severity of their dyspnea, which makes assessments difficult [8,12,13]. The inaccurate assessment of dyspnea severity is particularly problematic in end-stage COPD when patients are most affected by dyspnea [1]. However, their dyspnea is often assessed subjectively by physician observation only. There are several validated assessment tools for dyspnea, namely the modified Medical Research Council (mMRC) scale [14] and the COPD Assessment Test (CAT) [15]. The mMRC is a five-level scale of activity limitation due to dyspnea, while lacks the evaluation of self-limiting their living behaviors. Although the CAT includes a self-limiting living behaviors factor, it is a five-point Likert scale and is abstract. Physicians should discuss self-limiting behaviors with patients and evaluate the causes affecting them in a multifaceted manner, and intervene appropriately. Therefore, the lack of a simplified, specific and comprehensive patient-reported outcome measure remains a major challenge.

The unmet needs for dyspnea-related behavior and activity limitations in patients with COPD that have been described so far are similar to the measures of the frailty assessment for patients with COPD. Frailty is associated with geriatric syndromes that are recognized to represent a clinical state of physical, psychological, and social vulnerability and an increased risk for healthcare requirements [16]. Frailty is closely associated with COPD, which shares common risk factors such as aging, smoking, inflammation, and endocrine disorders [17]. As dyspnea increases, fatigue, muscle weakness, physical inactivity, and osteoporosis progress. These manifestations are similar to the symptoms of frailty. Once frailty progresses, the exacerbation of COPD, as a new physiological stressor, can result in increasingly worsening conditions ranging from disability and morbidity to death. The frequent coexistence of frailty and COPD seems to suggest a common underlying pathophysiological mechanism [18]. Actually, patients with COPD have a greater risk for frailty than patients without COPD [19,20]. Moreover, patients with comorbid COPD and frailty have even worse outcomes (exacerbations, hospitalization, mortality, and worse quality of life) [18,21,22,23,24,25]. Therefore, there is strong linkage of COPD, dyspnea and frailty. Although it is important to identify frailty in patients with COPD, the diagnosis of frailty requires comprehensive assessment and is not easily implemented in a busy daily clinical practice.

We developed a new dyspnea evaluation system that focuses on patients’ perceptions of dyspnea and their living disabilities, and named the evaluation tool “patient-reported outcome measures for dyspnea-related behavior and activity limitation (PROMs-D).” This study aimed to validate PROMs-D in patient with COPD and verify whether PROMs-D can identify frailty status.

## 2. Materials and Methods

### 2.1. Study Subjects

We recruited 128 consecutive outpatients with COPD who had been treated in the Yamaguchi University Hospital between May 2020 and July 2020. According to the GOLD diagnostic guidelines and criteria, COPD was defined as follows: all patients were ≥ 40 years old, had typical symptoms of COPD, such as chronic and progressive dyspnea or cough with sputum production, and had a postbronchodilator forced expiratory volume in 1 s (FEV1) to forced vital capacity ratio of <0.7. According to the recommendations of the American Thoracic Society/European Respiratory Society, pulmonary function was assessed using the CHESTAC-8800 DN type (Chest Ltd., Tokyo, Japan) [26]. Patients were excluded if they had experienced exacerbations during the four weeks prior to the study. Medical information and patient characteristics, including age, smoking status, disease severity, exacerbation history, current medication information, and the severity of comorbidities measured with the updated Charlson Comorbidity Index (CCI) [27] were obtained from the patients’ medical records. Exacerbations were defined as the use of systemic corticosteroids and/or antibiotics for worsening respiratory symptoms, with no evidence indicating an alternative diagnosis [4]. Body mass index (BMI) and the mMRC scale were measured for all participants when they were included in the study.

The study protocol and its amendments were approved by the local ethics committee of Yamaguchi Medical University (institutional review board no. H2020-112, approval date: 7 July 2020). The requirement for informed consent was waived by the ethics committee because no invasive procedure, intervention, or human samples were used in this observational study, and anonymity was secured. Our study was compliant with the Japanese Ethical Guidelines for Medical and Health Research Involving Human Subjects [28], which does not require informed consent from patients enrolled in studies that do not utilize human biological specimens. However, we provided opportunities for the subjects to opt out of the study by announcing the study information on bulletin boards in the hospital and the hospital website.

### 2.2. Patient-Reported Outcome Measures for Dyspnea-Related Behavior and Activity Limitation (PROMs-D)

We developed a new dyspnea evaluation system that focused on patients’ perceptions of dyspnea and views of their living disabilities, and called the system the “patient-reported outcome measures for dyspnea-related behavior and activity limitation (PROMs-D).” The PROMs-D consisted of the Activity-limit Dyspnea Scale (ADS) and Self-Limit Dyspnea Scale (SDS), which are categorized into nine levels: three levels by three levels (Table 1). The ADS focuses on activities that can’t be performed, while the SDS focuses on avoiding living behaviors. The PROMs-D is evaluated as a simple self-reporting yes/no survey that consists of five questions that focus on validation (No. 1), on activities that cannot be performed (No. 2 and 4), and on avoiding living behaviors (No. 3 and 5) (Table 2). For example, if the answer is “yes to 1–4, and 5 is no,” the outcome will be ADS Lv.2 and SDS Lv.1. The outcome can usually be obtained within one minute.

### 2.3. Definition of Frailty Using the Kihon Checklist

The Kihon Checklist (KCL) is a self-administered questionnaire developed by the Japanese Ministry of Health, Labor, and Welfare to predict functional decline in community-dwelling elderly [29]. The KCL consists of 25 ”yes/no” questions and divided into seven domains: (1) instrumental daily living activities (questions 1–5), (2) physical strength (questions 6–10), (3) nutritional status (questions 11–12), (4) oral function (questions 13–15), (5) social activities (questions 16–17), (6) cognitive function (questions 18–20), and (7) depressive mood (questions 21–25). The total KCL score, which is a sum of 25 answers, ranges from 0 (no frailty) to 25 (severe frailty). KCL has a wide application not only in Japan, but has also been translated into English, Brazilian Portuguese, Spanish, and Chinese and shown to be adequate for cross-cultural studies [29,30,31,32,33]. We categorized patient frailty status as robust (0–3), prefrail (4–7), or frail (8–25), as previously reported [34].

### 2.4. Statistical Analysis

Although no a priori sample size calculation was conducted, a convenience sample of about 130 subjects was selected based on previous studies [23,35]. Data are shown as median (interquartile range). The characteristics between the two groups were compared using Mann–Whitney U and Fisher’s exact tests. Spearman’s rank-order correlation coefficient was used to determine the correlation between two variables. Using a receiver operating characteristic (ROC) curve, we determined the cutoff points for identifying the predictive factors for frailty. The accuracy of each predictive factor was assessed using the area under the ROC curve (AUC). Statistical analyses were performed using JMP Pro ^®^, version 14.0.0 (SAS Institute, Inc., Cary, NC, US). A probability value of less than 0.05 was considered statistically significant.

## 3. Results

### 3.1. Patient Characteristics

The baseline characteristics of the 128 patients are shown in Table 3. The median age of the patients was 73 years and the median FEV1 was 78.3%. About 90% of the patients consisted of group A and B. Based on the total KCL score, we classified 30 (23.4%), 50 (39.0%), and 48 (37.5%) subjects as robust, prefrail, and frail, respectively.

### 3.2. Validation of the PROMs-D Questionnaire

When the answers to question 1 and questions 2–5 for each patient were validated, 20 (15.6%) of the patients were discordant. They answered that there are no activities that cannot be performed or avoiding living behaviors in question 1, while in questions 2–5 they answered that there are.

### 3.3. Distribution of the PROMs-D

**Figure 1:**
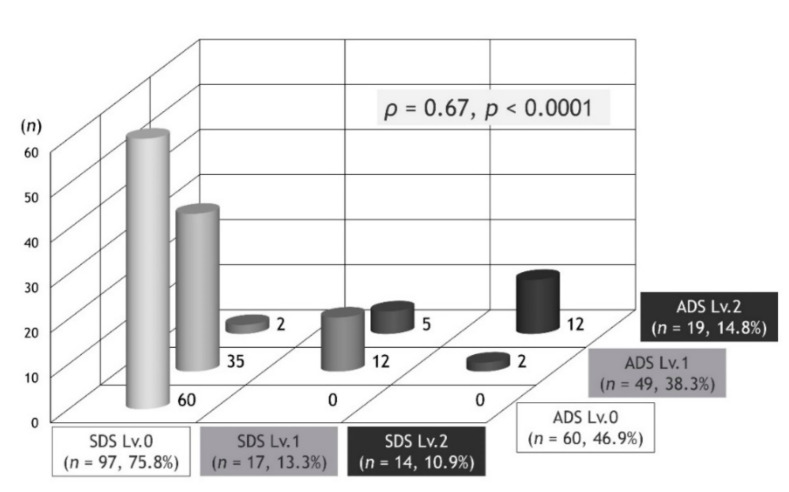
The distribution of the patient-reported outcome measures for dyspnea-related behavior and activity limitation (PROMs-D) combined with SDS and ADS. *ρ* refers to the Spearman’s rank-order correlation coefficient. Definition of abbreviations: SDS: Self-limit Dyspnea Scale; ADS: Activity-limit Dyspnea Scale.

The distribution of PROMs-D combined with SDS and ADS is shown in Figure 1. There was a positive correlation between SDS and ADS (*ρ* = 0.67, *p* < 0.001).

### 3.4. Relationship between Frailty Status and mMRC and PROMs-D

**Figure 2:**
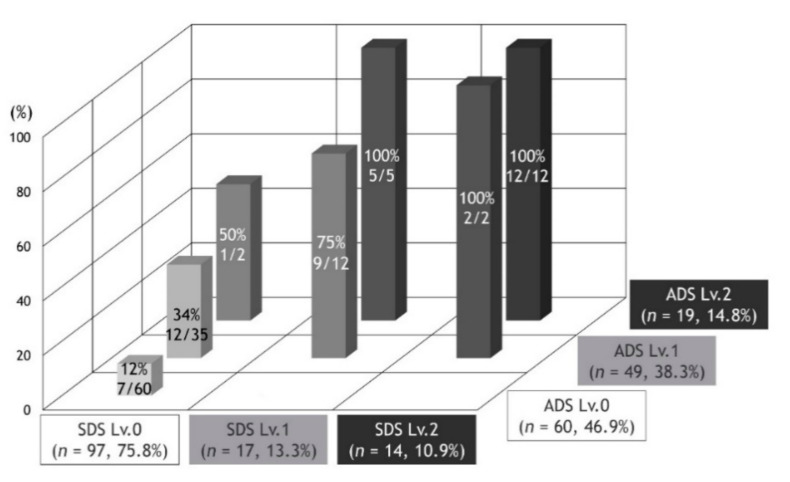
The prevalence of frailty stratified by PROMs-D. Definition of abbreviations: SDS: Self-limit Dyspnea Scale; ADS: Activity-limit Dyspnea Scale.

**Figure 3:**
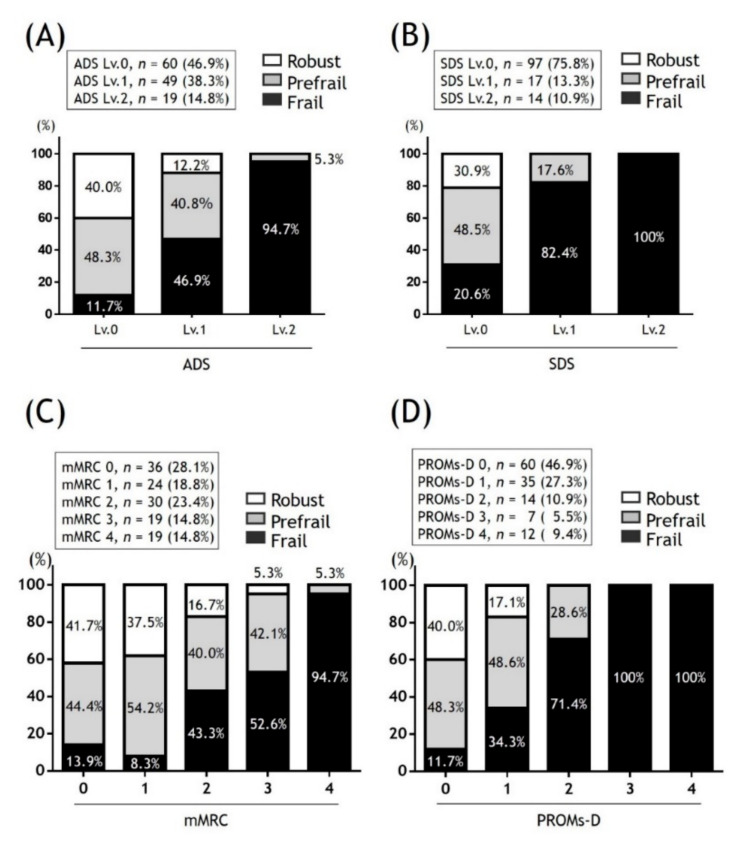
The proportion of frailty status according to the level of ADS (**A**); SDS (**B**); mMRC (**C**) and PROMs-D (**D**). Definition of abbreviations: ADS: Activity-limit Dyspnea Scale; mMRC: modified Medical Research Council; SDS: Self-limit Dyspnea Scale; PROMs-D: Patient-reported outcome measures for dyspnea-related behavior and activity limitation.

**Figure 4:**
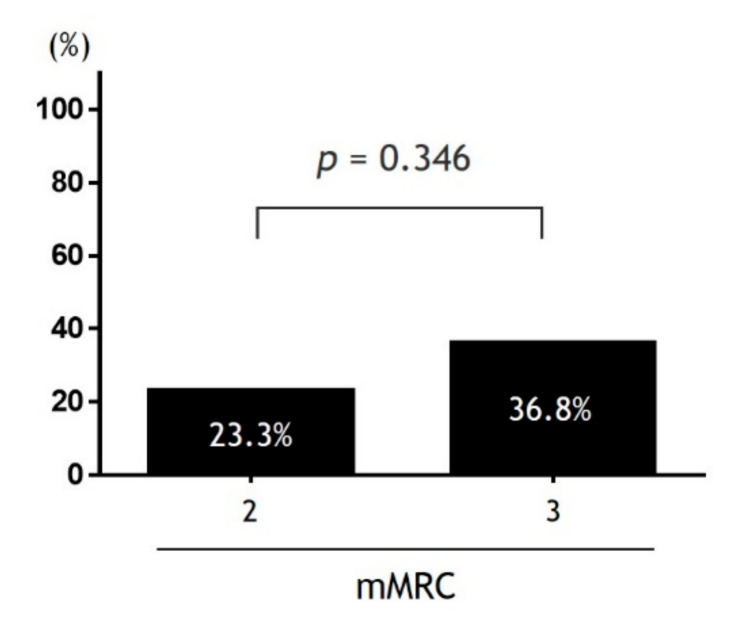
The prevalence of SDS ≥ 1 between the patients with mMRC 2 and 3. Definition of abbreviations: mMRC, modified Medical Research Council; SDS, Self-limit Dyspnea Scale.

The prevalence of frailty stratified by PROMs-D is shown in Figure 2. As the category moved from the lower left to the upper right, the prevalence of frailty increased. Based on this finding, we created a new variable named the PROMs-D score, which was calculated as the sum of ADS and SDS (Table 4). The range of scores was 0–4. The proportion of frailty status with respect to the ADS, SDS, mMRC, and PROMs-D score is shown in Figure 3. The higher the level of ADS, SDS, mMRC, and PROMs-D scores, the higher the proportion of frailty. We assessed the diagnostic ability of ADS, SDS, mMRC, and PROMs-D scores to identify frailty (Table 5). The AUCs for ADS, SDS, mMRC, and PROMs-D were 0.82, 0.78, 0.82, and 0.85, respectively. Moreover, we examined the prevalence of SDS ≥1 between the patients with mMRC 2 and 3 (Figure 4). There were no significant differences.

Different sensitivity, specificity, positive predictive value (PPV), negative predictive value (NPV), positive likelihood (LR+), and negative likelihood (LR−) values for each PROMs-D and mMRC cutoff value for predicting frailty are listed in Table 6 and Table 7. Compared with the diagnostic ability of PROMs-D score and mMRC grade to identify frailty, PROMs-D was more effective in stratification.

### 3.5. Comparison of the Frailty and Non-Frailty Groups of the Patients with PROMs-D Scores 0–1

In the cases of patients with PROMs-D scores of 0–1, 20% of patients were frail. Compared with the non-frailty group, the frailty group had a lower BMI (BMI < 21: 63.2% vs. 18.4%, *p* < 0.001) and a higher CCI score (CCI ≥ 5: 52.6% vs. 27.6%, *p* < 0.05).

### 3.6. Comparison of the Frailty and Non-Frailty Groups of the Patients with a PROMs-D Score of 2

In the cases of patients with a PROMs-D score of 2, 29% of patients were non-frail. Compared with the frailty group, the non-frailty group was younger (age < 65: 75% vs. 0%, *p* < 0.001).

## 4. Discussion

We have demonstrated the distribution of PROMs-D in patients with COPD; and although SDS showed a bias towards Lv.0, there was a positive correlation between SDS and ADS. After reviewing the relationship between PROMs-D and frailty status, PROMs-D was demonstrated to be a useful frailty screening tool for patients with COPD. Moreover, we propose the importance of capturing the troublesome nature of changing clothes and bathing due to dyspnea because it may trigger that physicians consider a new pharmacological and nonpharmacological COPD management.

Based on the simplicity and diagnostic ability of identifying frailty, PROMs-D could be used as the first step for frailty screening in patients with COPD. Previous studies have reported that St. George’s Respiratory Questionnaire (SGRQ) relates to frailty in patients with COPD [23]. However, because it takes too much time to measure, it is used as a research tool rather than in routine practice. The mMRC scale is a very simple and valid method for stratifying patients in terms of their risk of COPD exacerbation and airflow limitation [36]. It has been suggested to be useful as a frailty screening tool [22,35,37,38]. On the other hand, a recent study showed that there was little difference in frailty prevalence between mMRC 0 and 1 and between 2 and 3 [22], and our findings were consistent with this observation. According to the findings that focused on the prevalence of moderate or severe self-limitation between the patients with mMRC 2 and 3, there was no difference in this groups. Thus, we have identified that the assessment of mMRC alone may miss patients with self-limitation that require new therapeutic intervention such as self-management education, lung rehabilitation, and adequate assist use of short-acting β-agonist. It is possible to reduce the physician’s underestimation of self-limitation by capturing the troublesome nature of living behaviors due to dyspnea during the patient review even if the consultation time is limited. Therefore, it would be more useful for frailty screening to combine a three-level activity limitation assessment with the self-limitation and comorbidity assessment, rather than strictly dividing activity limitations into five levels. Our proposal makes sense because the mechanisms of dyspnea and the concept of frailty are multifactorial and encompass physiological, psychological, emotional, and social factors [16,39]. Of course, there are other easily applied frailty tests, such as the FRAIL scale, which that is 5 component patient-reported (yes/no) questions: Fatigue, Resistance, Ambulation, Illness, and Loss of weight [40]. The question on the FRAIL scale that applies to self-limitation is Fatigue; “How much time during the previous 4 weeks did you feel tired?”. Compared with the FRAIL scale, the SDS questions on the PROMs-D are highly specific recall questions. If physicians ask the patient’s self-limitation when using abstract questions, the patient may underestimate. Therefore, we believe that highly specific recall questions are more effective in identifying self-limitation than abstract questions. Furthermore, the SDS questions are very simple and take almost no time. If the answer to SDS questions is “yes”, physicians can share the patient’s specific self-limitation and consider interventions to resolve it. This is an advantage over other tools.

The frailty group in the patients with PROMs-D score 1–2 had a higher CCI score than the non-frailty group. Since comorbid conditions in COPD are closely related to respiratory symptoms, exacerbations, and physical inactivity, it is important to identify therapeutic targets for these unmet medical needs [25]. Several studies have reported that comorbidities in COPD are associated with an increased risk of frailty [21,22]. We realized the importance of focusing on comorbidities and considering the possibility of frailty, even in patients with COPD without self-limitations due to dyspnea.

**Figure 5:**
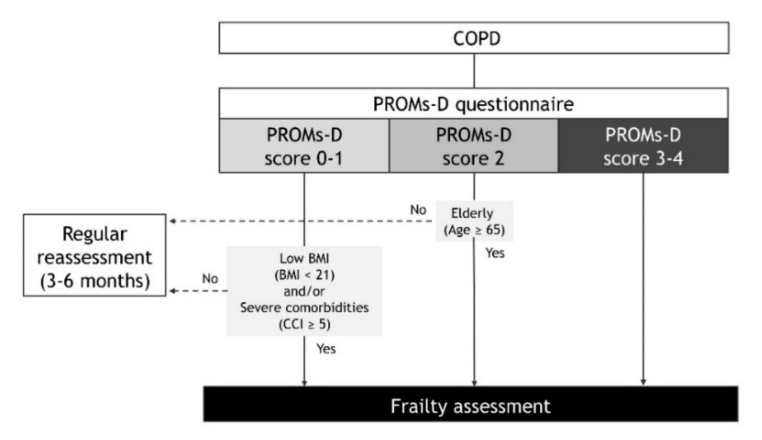
The strategy that consider frailty assessment according to the PROMs-D score in patients with chronic obstructive pulmonary disease (COPD). Definition of abbreviations: PROMs-D: Patient-reported outcome measures for dyspnea-related behavior and activity limitation.

There are various tools that assess frailty in elderly patients, with the Fried frailty phenotype (FFP) being the most widely used. FFP consists of five components (weakness, slowness, unintentional weight loss, exhaustion, and low physical activity) that classify individuals as robust, prefrail, or frail, depending on the number of components affected, respectively, zero, one or two, and three or more [41]. From the findings of the present study, we propose the following strategies in patients with COPD that consider frailty in daily clinical practice (Figure 5). In the case of PROMs-D scores of 0-1, although the probability of frailty is low, we recommend that frailty assessment may be considered when BMI is low (BMI < 21) and/or comorbidities are severe (CCI ≥ 5). In the case of a PROMs-D score 2, we suggest frailty assessment unless younger patients (age < 65 years). In the case of PROMs-D scores of 3–4, we strongly suggest that the frailty assessment should be performed immediately, due to the extremely high probability of frailty. In addition, it is necessary to routinely reassess PROMs-D after 3–6 months, even if the frailty assessment was not performed in patients with PROMs-D scores of 0–2. This is because patients with COPD also have a high prevalence of prefrailty, which is likely to move towards frailty.

The comprehensive management of patients with COPD with frailty that includes multidisciplinary teamwork and a community-based integrated care system, is crucial for the realization of a long healthy life expectancy society [42,43,44,45,46,47]. Since COPD is a very common disease, it is ideal for the frailty assessment to be comprehensively and easily assessed by primary care providers, pulmonary specialists, and other physicians. From this viewpoint, our proposal could contribute to improving the overall management of COPD.

In the present study, there was a positive correlation between SDS and ADS. In fact, all patients with ADS Lv.0 were SDS Lv.0, and the cohort with ADS Lv.2 had the highest number of patients with SDS Lv.2. However, at the time of ADS development, we assumed that there would be a significant number of patients with SDS Lv.1 and Lv.2. Against assumptions, SDS showed a bias towards Lv.0, and SDS Lv.0 accounted for three-quarters of the total number of subjects, with SDS Lv.0 being the most common among patients with ADS Lv.1. The level for troublesome changing of clothes and bathing due to dyspnea may refer to a higher level of self-limitation than what was estimated. This survey will continue to require validation and modifications for SDS. For example, it may be meaningful to capture the troublesome nature of certain living behaviors other than changing clothes and bathing.

The current study has some limitations. First, the study included a small number of subjects, and the patients were characterized by older age, lower BMI, male majority, and low prevalence of current smokers. Although the characteristics of patients in the present study are comparable with other studies in Japan [36,48,49], these patient characteristics may have influenced the findings, and there could be unmeasured confounders that could impact the results. Second, although we diagnosed frailty status using the KCL, there might be inconsistencies in frailty classification between various diagnostic tools. The prevalence of frailty was 37.5% of the subjects in the present study, which was higher than that of other studies [19], although a direct comparison is difficult due to the differences in both criteria used and populations or settings studied. Third, our study did not measure the CAT. Because the CAT covers a broad variety of the health status of COPD and is related to frailty in COPD patients [22,23,35,38], a demonstration of the relationship between PROMs-D and CAT is also needed. Fourth, based on validation of the PROMs-D questionnaire, there were a few discrepancies between the answers in question 1 and questions 2–5. There was no contradiction between the patients answering “Yes” to question 1 and “No” to questions 2–5. This was because the patients had activity limitations or self-limitations than were captured by questions 2–5. However, there was a discrepancy between the patients answering “No” to question 1 and “Yes” to any of questions 2–5. One reason for this discrepancy is that for about 30% of the patients, 2–5 were highly specific recall questions, whereas question 1 was a somewhat abstract. Since several reports recommended asking as specific questions as possible about dyspnea measurement [39,50], underestimation may occur unless the physician ask the patient specific questions about dyspnea that impacts daily living. Thus, we did not exclude the patients with discrepancies in the analysis. Anyway, the PROMs-D questionnaire may continue to need further improvement to solve these problems, and comparison with current standard assessment tools. Finally, various therapies and comorbidities may have influenced the findings. Although we evaluated comorbidities with the updated CCI, we did not evaluate other important comorbid conditions in COPD, such as obstructive sleep apnea and gastroesophageal reflux. Further large-scale studies are required to validate these results.

## 5. Conclusions

In this cross-sectional study, we analyzed the distribution of PROMs-D in patients with COPD and the relationship between PROMs-D and frailty status. Evaluation of dyspnea from the perspective of self-limiting living behaviors as well as activity limitation is useful for stratifying the frailty, so that PROMs-D could be used as the first step for frailty screening in patients with COPD. Moreover, we propose the importance of capturing the troublesome nature of changing clothes and bathing due to dyspnea, because it can provide an opportunity for physicians to consider new pharmacological and nonpharmacological COPD management. Although validation and modification studies are needed, the PROMs-D has great significance during the patient review, even if the consultation time is limited, and it is expected to be applicable to other respiratory diseases, such as ILDs.

## Figures and Tables

**Table 1:** Patient-reported outcome measures for dyspnea-related behavior and activity limitation.

Activity-limit Dyspnea Scale (ADS): Focusing on activities that cannot be performed.	
No or mild limitation (Lv.0)	Not applicable to the following items
Moderate limitation (Lv.1)	I walk slower than contemporaries on the level due to dyspnea
Severe limitation (Lv.2)	I can’t leave the house by myself due to dyspnea
Self-limit Dyspnea Scale (SDS): Focusing on avoiding living behaviors	
No or mild limitation (Lv.0)	Not applicable to the following items
Moderate limitation (Lv.1)	I feel changing clothes and bathing are troublesome due to dyspnea
Severe limitation (Lv.2)	I don’t change clothes or take a bath except when needed due to dyspnea

**Table 2:** PROMs-D questionnaire.

No.	Questions	Answer
1	Due to dyspnea, there are activities that can’t be performed or avoiding living behaviors.	Yes/No
2	I walk slower than contemporaries on the level due to dyspnea.	Yes/No
3	I feel changing clothes and bathing are troublesome due to dyspnea.	Yes/No
4	I can’t leave the house by myself due to dyspnea.	Yes/No
5	I don’t change clothes or take a bath except when needed due to dyspnea.	Yes/No

**Table 3:** Characteristics of the study subjects. (*n* = 128).

Variable	Value
Age (years)	73 (69–78)
Gender male	117 (91.2)
BMI (kg/m^2^)	22.7 (20.9–24.4)
Smoking status (never/ex/current)	5/112/11 (3.9/87.5/8.6)
mMRC grade (0/1/2/3/4)	36/24/30/19/19 (28.1/18.8/23.4/14.8/14.8)
Number of exacerbations (0/1/2)	110/6/12 (85.9/4.7/9.4)
GOLD (A/B/C/D)	60/56/0/12 (46.9/43.8/0/9.4)
FVC (L)	3.23 (2.63–3.78)
FVC (% predicted)	99.5 (85.2–113.5)
FEV1 (L)	2.06 (1.66–2.33)
FEV1 (% predicted)	78.3 (68.6–86.9)
FEV1/FVC (%)	63.7 (54.9–69.0)
Charlson comorbidity index (point)	3 (3–5)
Monotherapy of LAMA or LABA	28 (21.9)
Combination therapy of LAMA + LABA	98 (76.6)
ICS medication	48 (37.5)
Long-term oxygen therapy	17 (13.3)
KCL (point)	6 (4–11)
Frailty status (Robust/Prefrail/Frail)	30/50/48 (23.4/39.0/37.5)

Data are presented as median (interquartile range) or number (%). Definition of abbreviations: BMI, body mass index; mMRC, modified Medical Research Council; GOLD, Global Initiative for Chronic Obstructive Lung Disease; FVC, forced vital capacity; FEV1, forced expiratory volume in 1 s; LAMA, long-acting muscarinic antagonist; LABA, long-acting β-agonist; ICS, inhaled corticosteroid; KCL, Kihon Checklist.

**Table 4:** Scoring for the PROMs-D score.

Activity-Limit Dyspnea Scale (ADS)	
0 point	Not applicable to the following items
1 point	I walk slower than contemporaries on the level due to dyspnea
2 point	I can’t leave the house by myself due to dyspnea
Self-limit Dyspnea Scale (SDS)	
0 point	Not applicable to the following items
1 point	I feel changing clothes and bathing are troublesome due to dyspnea
2 point	I don’t change clothes or take a bath except when needed due to dyspnea
PROMs-D score = ADS + SDS. (range of score: 0–4)	

Definition of abbreviations: PROMs-D: Patient-reported outcome measures for dyspnea-related behavior and activity limitation.

**Table 5:** Diagnostic ability in ADS, SDS, mMRC, and PROMs-D to identify frail.

	AUC (95% CI)	Sensitivity	Specificity	Cut off Value
ADS	0.82 (0.75–0.89)	85%	66%	Lv.1
SDS	0.78 (0.71–0.85)	58%	96%	Lv.1
mMRC	0.82 (0.74–0.90)	85%	67%	2
PROMs-D score	0.85 (0.78–0.92)	60%	95%	2

Definition of abbreviations: mMRC, modified Medical Research Council; AUC, area under the curve; CI, confidence interval.

**Table 6:** Accuracy of PROMs-D score in predicting for frailty.

PROMs-D Score	Sensitivity (%)	Specificity (%)	PPV (%)	NPV (%)	LR+	LR−
1	85.4	66.3	60.3	88.3	2.53	0.22
2	60.4	95.0	87.9	80.0	12.08	0.42
3	39.6	100.0	100.0	73.4	Inf	0.60
4	25.0	100.0	100.0	69.0	Inf	0.75

Definition of abbreviations: PROMs-D: Patient-reported outcome measures for dyspnea-related behavior and activity limitation; PPV, positive predictive value; NPV, negative predictive value; LR+, positive likelihood ratio; LR−, negative likelihood ratio.

**Table 7:** Accuracy of mMRC grade in predicting for frailty.

mmrc Grade	Sensitivity (%)	Specificity (%)	PPV (%)	NPV (%)	LR+	LR−
1	89.6	38.8	46.7	86.1	1.46	0.27
2	85.4	66.3	60.3	88.3	2.53	0.22
3	58.3	87.5	73.7	77.8	4.67	0.48
4	37.5	98.8	94.7	72.5	30.0	0.63

Definition of abbreviations: mMRC, modified Medical Research Council; PPV, positive predictive value; NPV, negative predictive value; LR+, positive likelihood ratio; LR−, negative likelihood ratio.
